# The complete mitochondrial genome of *Pontia edusa* (Lepidoptera: Pieridae)

**DOI:** 10.1080/23802359.2020.1844091

**Published:** 2021-01-11

**Authors:** Ju-Ping Wang, Yun-Fei Peng, Tian-Wen Cao, Miao Zhang

**Affiliations:** Shanxi Insect Herbarium, College of Plant Protection, Shanxi Agricultural University, Taiyuan, China

**Keywords:** *Pontia edusa*, complete mitogenome, Lepidoptera

## Abstract

The complete mitochondrial genome of *Pontia edusa* was sequenced and analyzed in the study. The length of the complete mitogenome is 15,125 bp, including 37 genes and a control region. Twelve genes start with typical ATN, but *COI* gene initiate with a CGA codon. Twelve of 13 PCGs have a complete stop codon TAA or TAG except for *COI* has an incomplete stop codon T––. Twenty-one of the 22 tRNAs have a typical clover-leaf secondary structure. The *tRNA^Ser(AGN)^* gene lacked the DHU loop. Bayesian analyses highly support the monophyly of Pieridae. In Pieridae, *P. edusa* is subordinate to the Pierinae clade.

*Pontia edusa* (Fabricius, 1777) is a member of the Pontia Fabricius of the subfamily Pierinae (Lepidoptera: Pieridae). The larva of *P. edusa* mainly harms Reseda, Turritis, Sisymbrium, Sinapis and Alyssums plants, such as *Reseda odorata*, *Turritis glabra*. The species is distributed in Europe, northwestern India, Siberia, North Africa to Ethiopia and most regions of China (Wu 2001). In this study, the complete mitogenome sequence of *P. edusa* was determined and described. The genome assembly is useful for broader research in the field of molecular ecology, systematics and phylogenetics. Meanwhile, the study also provided valuable genetic information for the identification and classification of the species more correctly. The Voucher specimen number is YF20160816 and the sequence data was deposited in GenBank under accession no. MK252290. This genome resource would be used by Shanxi Key Laboratory of Integrated Pest Management in Agriculture and Shanxi Insect Herbarium.

The adult specimen of *P. edusa* was collected from ShuoZhou City, Shanxi Province, China (39.45 N, 112.36E) in July 2016. Some specimens were preserved by spreading the wings in the Shanxi Insect Herbarium, College of Plant Protection, Shanxi Agricultural University and their numbers are YF20160817-20160827. The other specimens were conserved in anhydrous ethanol at −20 °C in the laboratory. Total genomic DNA was extracted from thoracic muscle of a single adult using the OMEGA Insect DNA Kit. An Illumina TruSeq library was prepared with an insert size of 350 bp and sequenced using the Illumina NovaSeq 6000 platform with 150 bp paired-end reads. Raw reads were checked by FastQC 0.11.3 (Andrews 2010), and low-quality reads were filtered by Trimmomatic (Bolger et al. 2014) and Prinseq (Schmieder and Edwards 2011), respectively. A total of 6 Gb clean data were obtained and assembled by IDBA-UD (Peng et al. 2012), and then the mitochondrial genome sequences were identified through the Geneious 10.1.3 (http://www.geneious.com/). The PCGs and two rRNA genes were confirmed by alignment with homologous genes from closely related species. The 22 tRNA genes were annotated using tRNAscan-SE version 2.0.2 (Lowe and Chan 2016).

The complete mitochondrial genome of *P. edusa* is 15,125 bp in size, containing 13 protein-coding genes, 22 tRNA genes, two rRNA genes, and a control region. Twenty-three genes are encoded on major strand (J-strand), the other 14 genes are found in the minor strand (N-strand). The gene order and orientation are identical to the sequenced lepidopteran mitogenomes (Cao et al. 2013; Wang et al. 2013, 2016).

The *P. edusa* mitogenome is biased toward AT content at 79.91%. The nucleotide compositions are 39.91% of A, 40.00% of T, 12.23% of C, 7.85% of G. Twelve genes start with typical ATN (five with ATG, two with ATA, two with ATC, three with ATT), but *COI* gene initiate with a CGA codon which was observed in most lepidopteran insects (Kim et al. 2014; Song et al. 2016; Zhang et al. 2019). Twelve of 13 PCGs harbor the usual complete stop codon TAA or TAG, and the remaining *COII* gene possess the incomplete stop codons T.

The *P. edusa* mitogenome has the typical 22 tRNA set. The 22 tRNAs were interspersed throughout the whole mitogenome and ranged from 60 to 71 nucleotides. Twenty-one of the 22 tRNAs have a typical clover-leaf secondary structure. The *tRNA^Ser(AGN)^* gene lacked the DHU loop. This incomplete *tRNA^Ser^* (60 bp) structure has been found in many lepidopteran insects (Hong et al. 2009; Zhang et al. 2012). The *rrnL* is located between *tRNA^Leu(CUN)^* and *tRNA^Val^*, the lengths is 1317 bp and the A + T content is 84.05%. The *rrnS* is located between *tRNA^Val^* and the control region, the lengths is 774 bp and the A + T content is 85.15%. The control region is located between *rrnS* and *tRNA^Met^* and is 373 bp long with a significant AT bias (93.03%).

The 13 protein-coding genes of 21 known Pieridae and the other 4 Lepidoptera taxa were concatenated to reconstruct the phylogenetic relationships by Bayesian analysis (Figure 1). The sequences were aligned with the Mega 7.0 software (Kumar et al. 2016). The aligned datasets were analyzed with the Markov Chain Monte Carlo (MCMC) algorithm under GTR + I + G model using MrBayes3.1.2 (Huelsenbeck and Ronquist 2001). The *Hyphantria cunea* (NC_014058) and *Lymantria dispar* (NC_012893) were selected as outgroups. Bayesian analyses highly support the monophyly of Pieridae (PP =1.00). In Pieridae, the relationship of the subfamilies is: Dismorphiinae+ (Pierinae + Coliadinae). The newly sequenced species *P. edusa* is subordinate to the Pierinae clade.

**Figure 1.**
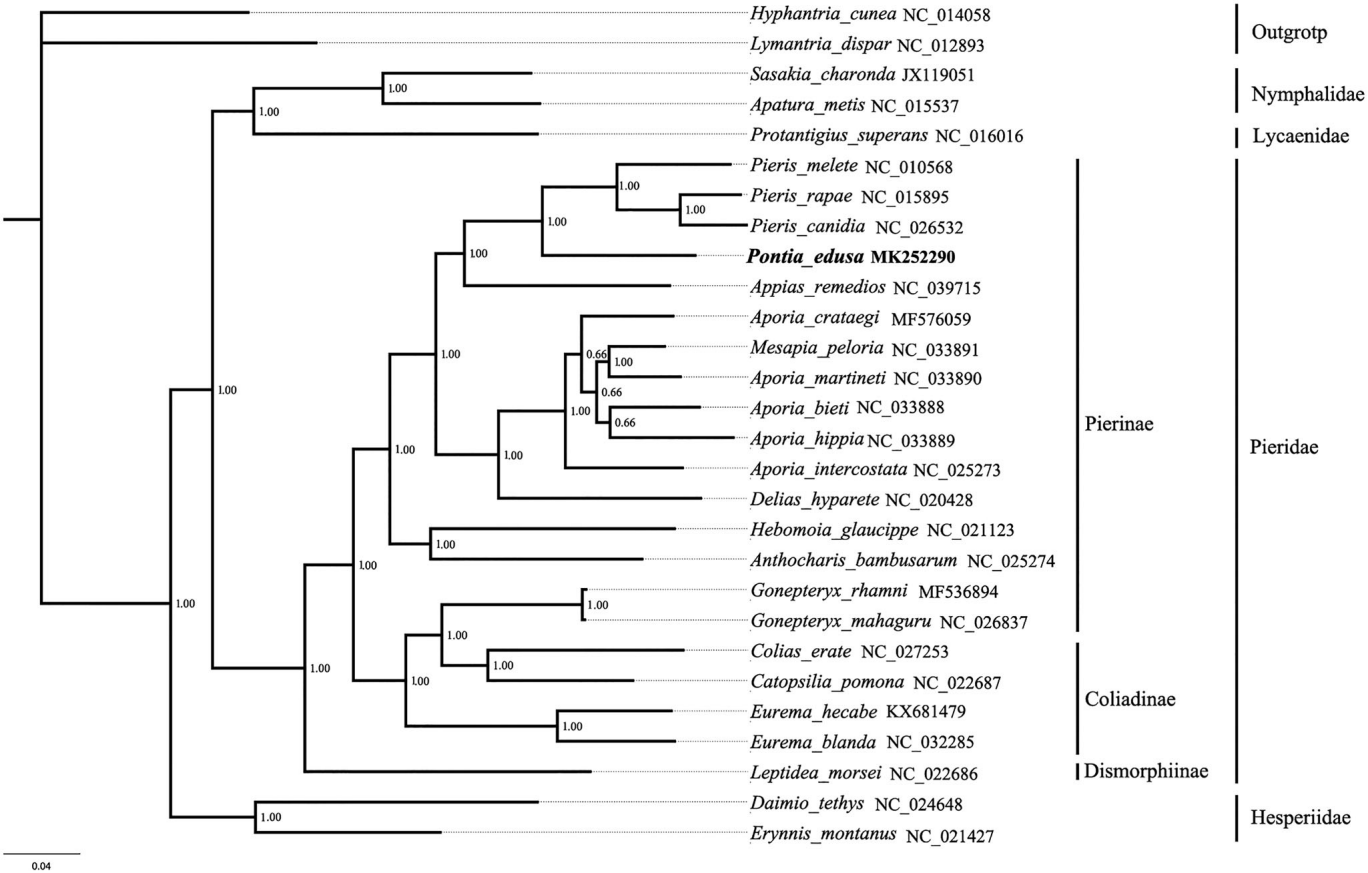
Bayesian phylogenetic tree based on 13 protein-coding genes of the mitochondrial genome sequences of 21 Pieridae species.

## Data Availability

The genome sequence data that support the findings of this study are openly available in GenBank of NCBI at https://www.ncbi.nlm.nih.gov under the accession no. MK252290. The associated BioProject, SRA, and Bio-Sample numbers are PRJNA664249, SRR12806058, and SAMN16205026 respectively.
